# Prognostic Impact of a Routine Six-Month Exercise Stress Test after Complex Left Main Bifurcation Percutaneous Intervention

**DOI:** 10.3390/diagnostics14010059

**Published:** 2023-12-26

**Authors:** Gianluca Rigatelli, Marco Zuin, Giuseppe Marchese, Ervis Hiso, Giulio Rodinò, Loris Roncon, Giampaolo Pasquetto

**Affiliations:** 1 Interventional Cardiology Unit, Division of Cardiology, Aulss6 Ospedali Riuniti Padova Sud, 35043 Monselice, Italy; giuseppe.marchese@aulss6.veneto.it (G.M.); ervis.hiso@aulss6.veneto.it (E.H.); giulio.rodino@aulss6.veneto.it (G.R.); giampaolo.pasquetto@aulss6.veneto.it (G.P.); 2 Department of Specialistic Medicine, Division of Cardiology, Rovigo General Hospital, 45100 Rovigo, Italy; 3 Department of Translational Medicine, University of Ferrara, 44121 Ferrara, Italy; zuinml@yahoo.it

**Keywords:** stent, left main bifurcation, exercise stress test

## Abstract

The prognostic value of exercise stress test after complex left main (LM) coronary artery bifurcation (LM) stenting has been poorly investigated. To partially fill this gap in knowledge, we retrospectively analyzed the procedural and medical data of consecutive patients referred to our center for complex LM bifurcation disease between January 2008 and May 2018 who were treated using either single- or dual-stenting techniques. The prognostic impact of an exercise stress test, performed 6 months after the coronary intervention, was evaluated in 502 patients (316 males, mean age 70.3 ± 12.8 years, mean Syntax score 31.6 ± 6.3). At follow up after a mean of 37.1 ± 10.8 months (range 22.1–47.3 months), the target lesion failure (TLF) rate was 10.1% while stent thrombosis and cardiovascular mortality were 1.2 and 3.6%, respectively. A positive exercise stress test was detected at 6-month follow up in 42 out of 502 patients (8.4%); the incidence of a significant restenosis was 7.6% (*n* = 38). Patients with a negative exercise stress test at 6-month follow up had higher freedom from TLF and improved survival compared to those with a positive exercise stress test.

## 1. Introduction

In selected patients, due to continuous interventional and technological advances, complex left main (LM) bifurcation percutaneous interventions (PCI) have become safer and more widely used as an alternative to standard aorto-coronary bypass surgery (CABG) [1,2,3,4]. As suggested in the current recommendations published by the European Bifurcation Club (EBC) [5] and according to recent randomized clinical trials (RCTs) and meta-analysis results [6], crossover provisional stenting remains the gold-standard technique for the percutaneous interventional management of LM bifurcation disease. However, in recent years, the role of double-stenting techniques in distal bifurcation LM disease has gained increasing interest [7], considering the positive results provided by different large analyses [8]. 

Although the current guidelines for exercise stress testing [9] state that routine early exercise stress test after PCI is not indicated, because of the increasing number of complex PCI cases worldwide the need for detecting patients with subclinical ischemia after complex and demanding PCI of LM bifurcation has increased, and thus would still be worthy of investigation. As a matter of fact, the role of stress tests during follow ups with these patients has not yet been completely clarified in relation to the different techniques used. Indeed, symptom recurrency and/or silent ischemia detected using nuclear stress tests as well as stress echocardiography and exercise tests are used differently worldwide to evaluate the need for angiographic reevaluation. These differences are partially due to specific institutional protocols, availability of different techniques, patient’s comorbidities, and relative performance status, as well as related costs. Although these are of only limited significance in patients with compete bundle branch blocks and are unsuitable for frailty or physical constraints, the exercise stress test has the advantage of being easy, widely available, and with limited associated costs. The aim of this study is to retrospectively evaluate the prognostic role of routine exercise stress test after 6 months from the index revascularization in patients having a complex LM stenting treated with different techniques, including crossover provisional stenting, culotte, T-and-protrusion (TAP), and nano-inverted-T (NIT) stenting.

## 2. Materials and Methods

### 2.1. Patients Enrolled

We retrospectively analyzed the procedural and medical data of consecutive patients referred to our center for complex LM bifurcation disease, treated using crossover provisional stenting, culotte, T-and-protrusion (TAP), and nano-inverted-T (NIT) stenting [10] between 1 January 2008 to 1 May 2018 due to contraindications and/or refusal of surgical treatment. Traditional cardiovascular risk factors, the Canadian Cardiovascular Score class (CCS), EuroSCORE II [11], SYNTAX score [12], MEDINA classification [13] as well as pre-and postprocedural angiographic characteristics were revised and analyzed by the local heart team, which included a clinical cardiologist, a cardiac surgeon, and an interventional cardiologist. Agreement was reached in 98.8% of cases; any discrepancy was discussed and resolved by consensus between tow interventionalists with 20 years of experience in the treatment of LM bifurcation (G.R and G. P.). Written informed consent to the indexed procedure was obtained from all patients before intervention. 

Inclusion criteria for LM PCI were as follows: (i) patients presenting with silent ischemia or stable or unstable angina; (ii) the involvement of distal LM bifurcation lesion (Medina 1,1,1 or 0,1,1), with >50% diameter stenosis (DS) of both the ostial left anterior descending (LAD) and left circumflex (LCx) coronary arteries; (iii) visual estimation and confirmed using fractional flow reserve (FFR) or intravascular ultrasound (IVUS). Conversely, exclusion criteria were as follows: patients who developed an intraprocedural ST-elevation myocardial infarction (STEMI) with vessel occlusion as complication of an elective procedure, those previously treated with CABG, and if they present in-stent restenosis (ISR) or any clinical condition that could interfere with medications compliance or follow up. Finally, all patients who were not suitable for exercise stress test were excluded from the analysis, such as those with complete left bundle branch block (LBBB) or right bundle branch block (RBBB), frailty or physical constraints enabling to perform the test, and those who died before the 6-month exercise stress test.

### 2.2. Definitions 

Target lesion failure (TLF) was defined as the composite of cardiovascular death, target vessel myocardial infarction (TVMI), and clinically driven target lesion revascularization (TLR). Cardiovascular mortality from cardiac causes was defined as any death from clear cardiac causes. Protocol-defined periprocedural acute myocardial infarction (AMI) was defined as coronary intervention-related myocardial infarction (MI), defined by an elevation of Troponin (cTn) values more than five times the 99th percentile URL in patients with normal baseline values. In patients with elevated preprocedure cTn and in whom the cTn level was stable (≤20% variation) or falling, the postprocedure cTn must rise by >20%. However, the absolute postprocedural value must still be at least five times the 99th percentile URL. Additionally, one of the following elements was required: new ischemic electrocardiographic changes; development of new pathological Q waves, or imaging evidence of new loss of viable myocardium or new regional wall motion abnormality in a pattern consistent with an ischemic etiology. Angiographic findings consistent with procedural flow-limiting complications such as coronary dissection, occlusion of a major epicardial artery or a side branch occlusion/thrombus, disruption of collateral flow, or distal embolization have been defined as Type 4a MI [14]. Spontaneous MI was defined as detection of a rise and/or fall of cTn values with at least one value above the 99th percentile URL and with at least one of the following: symptoms of acute myocardial ischemia; new ischemic ECG signs; development of pathological Q waves; imaging evidence of new loss of viable myocardium or new regional wall motion abnormality in a pattern consistent with an ischemic etiology; or identification of a coronary thrombus via angiography, including intracoronary imaging or by autopsy (Type 1 MI) [14]. Stent thrombosis (ST) was classified according to the Academic Research Consortium (ARC) definitions as definite, probable, or possible and as early (0–30 days), late (31–360 days), or very late (>360 days). In-stent restenosis (ISR) was evaluated by quantitative coronary angiography (QCA) and eventually FFR if the luminal narrowing was <70% and classified as focal (<10 mm long), diffuse (>10 mm long), proliferative (>10 mm long and extending outside the stent edges), or totally occluded [15]. Complex LM bifurcation lesion was defined according to the DEFINITION (Definitions and Impact of Complex Bifurcation Lesions on Clinical Outcomes After Percutaneous Coronary Intervention Using Drug-Eluting Stents) study [16].

### 2.3. Interventional Protocol and Techniques 

A 6F right radial approach has been used whenever possible. During PCI, patients were anticoagulated with unfractionated heparin (a bolus of 40 U/kg and additional heparin to achieve an activated clotting time of 250–300 s). Choice of stenting techniques was at operator choice and included crossover provisional stenting, culotte, T-and-protrusion (TAP), and nano-inverted-T stenting. Patients could receive the Orsiro (BiotronikInc, Bulach, Switzerland), Xience (Abbot Inc., Santa Clara, CA, USA) and Promus Premiere (Boston Scientific Inc., Mantick, Fremont, CA, USA) or the Onyx Resolute (Medtronic Inc., Galway, Ireland) stents basing the diameter of the main vessel stent using Finet’s law [17] or preferably IVUS measurements, which was recommended in all enrolled patients whenever possible depending on availability. Additional significant lesions in other vessels were treated with staged procedures using a routine last generation DES. In patients with acute coronary syndrome, a twelve-month dual antiplatelet therapy (DAPT) was administered as per current international guidelines.

### 2.4. FFR and IVUS Protocol

FFR evaluation was performed using a Pressure-Wire X device (Abbot Medical, Plymouth, MN, USA) and intracoronary bolus injection of Adenosin with a dilution of 12 mg in 250 mL of NaCl solution (6–8 mL per run). Specifically, a mean cut off of <0.79 on at least three runs was considered significant. Intravascular ultrasound (IVUS) examination was performed routinely following current recommendations using the 3F Opticross coronary IVUS catheter (Boston Scientific, Fremont, CA, USA) and an automatic pullback system (0.5 mm/s). On-line ultrasound assessment was performed in diastole. IVUS images were recorded after administration of 100–200 mg of nitro-glycerine. A segment of 0.5 mm proximally and distally the lesion/stent was analyzed using motorized transducer pullback. IVUS images were interpreted by the treating physician and at least one experienced IVUS technician.

### 2.5. Exercise Test Protocol 

A cycle ergometer stress test was performed in all patients enrolled using a bicycle ergometer with a stepwise increment of 20 W every minute to reach 85% or more of the maximal age-predicted heart rate (Bruce’s protocol) [18,19]. Patients had to discontinue β-blockers, calcium antagonists, and nitrates for 24 h before testing. The ECG was monitored continuously throughout the procedure as well as during recovery to assess for arrhythmias or ischemic ST-segment deviations. The exercise stress test was discontinued at maximal stress (95% of the maximal cardiac frequency calculated by age), fatigue, or due to the occurrence of at least 2 mm of ST-segment depression or at least 1 mm ST-segment elevation (in a non-Q wave lead) in two continuous leads, exertional hypotension, chronotropic incompetence, worsening chest pain, or ventricular arrhythmias. The result of the exercise ECGs was interpreted as negative, positive, or inconclusive [20].

### 2.6. Follow Up

Per institutional protocol, follow up was conducted by physical examination and standard 12-lead electrocardiogram at 1, 6, and 12 months and then yearly. Transthoracic echocardiography (TTE) was scheduled at 6 months and then yearly. Exercise tests were conducted at 6 months and thereafter on the referral physician’s discretion. IVUS-guided angiography was performed only at the time of additional vessel treatment or based on clinical symptoms or instrumental evidence of myocardial ischemia on exercise or nuclear stress test. Post-discharge survival status was obtained from the Municipal Civil Registries. Information on occurrence of acute MI or repeated interventions at follow up was collected by consulting our institutional electronic database and by contacting referring physicians and institutions and all living patients.

### 2.7. Statistical Analysis 

Continuous variables were presented as mean ± standard deviation while categorical data were summarized as frequencies and relative percentages. For continuous variables, normal distribution was evaluated using Kolmogorov–Smirnov test. Differences among groups were analyzed using Student’s *t*-test or one-way analysis of variance (ANOVA) followed by post hoc Bonferroni test. Kaplan–Meier analysis was applied to represent the freedom from TLF over the follow-up period in patients with negative or positive exercise stress test at 6 months. Statistical significance was defined as *p* < 0.05. Statistical analyses were performed using SPSS package version 20.0 (SPSS, Chicago, IL, USA).

## 3. Results

### 3.1. Population and Procedures

Over the study period, 754 patients received a complex LM bifurcation PCI. After revision and application of inclusion and exclusion criteria, 502 patients (316 males, mean age 70.3 ± 12.8 years, mean Syntax score 31.6 ± 6.3) were included in the final analysis. Specifically, 252 patients did not meet the study criteria: 60 patients for a complete RBBB, 89 for a complete LBBB, 36 patients for STEMI <24 h the procedure, and 67 due to physical constraints or frailty preventing them from performing the test or due to loss at follow up before 6 months. The clinical characteristics and comorbidities of the population enrolled are presented in Table 1. Coronary angiography evidenced a mean angle between LM and LCx of 64.8 ± 20.7° (range 17 to 91 degrees). Lesion characteristics are shown in Table 2. IVUS was performed in 76.7% (*n* = 132/172), 29.8% (*n* = 51/171), 16.4% (*n* = 10/61), and 37.7% (*n* = 37/98) of patients stented using NIT, crossover, T or TAP stenting, and culotte, respectively.

### 3.2. PCI Outcomes

Clinical follow up was available for all patients, as per inclusion criteria. At follow up after a mean of 37.1 ± 10.8 months (range 22.1–39.3 months), the overall TLF rate was 10.1% (6.4% in the crossover group, 16.4% in the T/TAP group, 16.3% in the culotte group, and 5.9% in the NIT group). Cardiovascular mortality rate was 3.6% (*n* = 18) while stent thrombosis occurred in 1.2% (*n* = 6) of cases. Clinically driven angiographic follow up was available in 135 patients (26.8%), at a mean time after the procedure of 7.8 ± 0.7 months, which showed a significant restenosis in 38 patients (clinically restenosis 7.6%), predominantly located at LCx ostium or within 5 mm from the ostium in 32 patients (84.2%) or in the LAD in the other 6 patients (15.8%). A re-PCI with a drug-coated balloon was applied in 29 patients, while in 9 patients an additional stent was implanted.

### 3.3. Prognostic Impact of 6-Month Exercise Test

**Figure 1:**
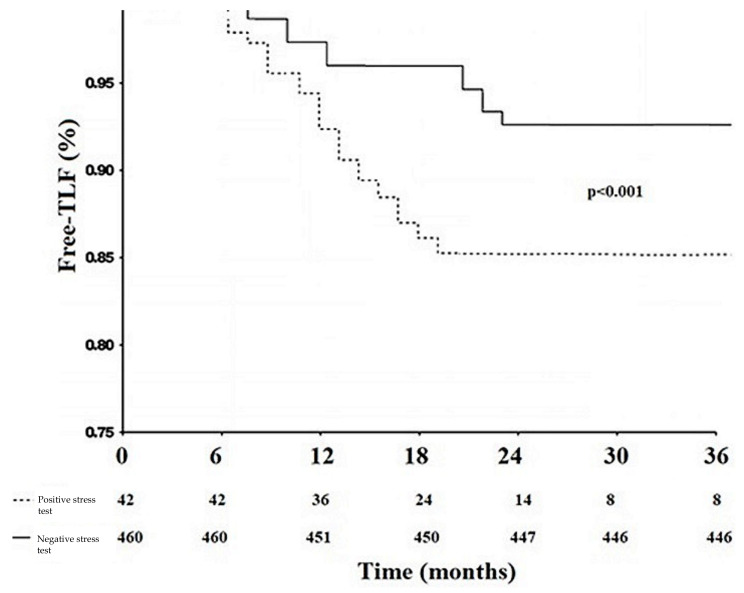
Kaplan–Meier of TLF free survival during the follow up (started six months after the index procedure): comparison between positive and negative six-month exercise stress tests.

A positive exercise stress test at a 6-month follow up was observed in 42 out of 502 patients (8.4%) and correlated with a significant angiographic restenosis 7.6% of cases (*n* = 38) (4.1% in the crossover group, 16.4% in T or TAP group (16.4%), 15.3% in the culotte group, and 3.5% in the NIT group). The exercise stress test patterns are shown in Table 3. In particular, patients treated using a single-stent strategy more frequently had a negative stress test (*p* = 0.01). Conversely, an ST-segment elevation in lead aVR > 1 mm (*p* = 0.04) or a ST-segment depression from V3 to V6 (*p* = 0.01) were more common in the double-stent strategy group. Stress test characteristics in the double-stent strategy group of patients are shown in Supplementary File S1. The distribution of clinical, anatomical, and procedural variables in patients with positive and negative 6-month exercise stress tests is presented in Table 4. Patients with a negative 6-month exercise stress test were associated with higher freedom from TLF compared to those with positive exercise stress tests (Figure 1).

## 4. Discussion

The present analysis has evidenced that a 6-month exercise stress test may detect a significant proportion of LM bifurcation stented patients who developed TLF. 

Although the exercise ECG test is not widely suitable for detecting stable CAD due to well-known limitations such as the ageing and increasingly overweight population. The exercise ECG test is the most available method in non-invasive CAD diagnostics with a sensitivity of 70–77% and a specificity of 65–80% in the general population [21]. However, in women, the sensitivity of the exercise ECG test is only 60% (54–68%) and its specificity is 70% (64–75%) [22,23]. In previous meta-analyses, based on patients with and without previous MI, the exercise stress test has shown a predictive accuracy ranging between 69 and 73%, as well as a sensitivity and a specificity ranging between 67 and 68% and 72 and 77%, respectively, ref. [24] for CAD diagnosis.

Past guidelines have recommended that routine functional testing after PCI should be performed only in select high-risk patients with decreased left ventricular function, multiple-vessel disease (VD), proximal LAD disease, previous aborted sudden cardiac death, diabetes mellitus, and suboptimal PCI results [25]. Indeed, evidence is lacking on the clinical utility of early exercise testing after PCI in the stenting era, especially after drug-eluting stent implantation and after complex LM interventions. The initial data from the Routine versus Selective Exercise Treadmill Testing after Angioplasty (ROSETTA) registry evidenced that routine functional testing after percutaneous transluminal coronary angioplasty is associated with reduced frequency of follow-up clinical events, including acute coronary syndrome (ACS) and death [26]. Similar findings have been reported both by Babapulle et al. [27] and Eisenberg et al. [28]. More recently, Cho et al. [29] investigated the role of early exercise stress testing in patients with single- or multivessel disease, demonstrating that early assessment after PCI might be helpful for predicting clinical outcomes in subjects with single-vessel disease and residual SYNTAX score ≤ 8.

Conversely, our study suggested that a traditional 6-month exercise stress test, based on Bruce’s protocol, can identify patients with TLF. Regarding the LM PCI technique used, our results confirmed that a single-stent strategy is associated with less positive exercise tests but also that among the double-stent strategies, the NIT seems to have the most beneficial effect, as demonstrated by the lower rate of positive exercise stress tests, TLF incidence, and CV mortality rate [30]. 

Obviously, beyond the technique used, extensive use of IVUS [31], proper sized stent platforms [32], and correct selection of the vascular approach and sheath size [33] all help to equalize the results of PCI to those of CABG.

From our study, since unfavorable events are more frequent in patients after PCI LM than after CABG [34,35,36], treadmill testing seem to be clinically relevant; ideal candidates are middle-aged patients of normal weight or mildly obese and without articular pathology.

### Limitations

Our study has obviously several limitations. Firstly, the retrospective, single-center, and non–randomized fashion of this study limited the accuracy and generalizability of our results. Secondly, the impact of different stents used over the study period, which have different geometrical and rheological properties, may have affected the final results. Thirdly, the different use of IVUS among the various subsets of patients might have contributed to different outcomes. Fourthly, the retrospective nature of our study did not allow investigation of the role of alternative stress tests either to evaluate the proportion of patients who were unable to undergo an exercise stress test or who were lost at follow up. Finally, multiple Cox regressions were not performed due to the limited number of events during the follow up. Nevertheless, we believe that the size of our patient sample and the length of the follow up could somewhat overcome these intrinsic limitations.

## 5. Conclusions

Our study suggests that a simple routine 6-month exercise test after complex bifurcation LM PCI may be useful to detect clinical restenosis and to prognosticate 3-year outcomes.

## Figures and Tables

**Table 1:** Demographic and clinical characteristics of the analyzed cohorts of patients. LVEF: left ventricular ejection fraction; CCS: Canadian class score; TIA: transient ischemic attack; HF: heart failure; CKF: chronic kidney failure; COPD: chronic obstructive pulmonary disease; PAD: peripheral artery disease; MI: myocardial infarction; N-STEMI: non-ST segment elevation myocardial infarction; STEMI: ST elevation myocardial infarction. * *p* < 0.05 NIT vs. crossover.

	Crossover *n* = 171	T or TAP *n* = 61	Culotte *n* = 98	NIT *n* = 172	*p*
Age (years)	68.3 ± 9.1	69.1 ± 10.3	71.9 ± 11.7	70.3 ± 12.8	0.60
Male	91 (53.1)	34 (55.7)	50 (51.0)	101 (58.7)	0.72
Obesity	24 (14)	11 (18.1)	16 (16.3)	27 (15.9)	0.67
Arterial hypertension, *n* (%)	95 (55.6)	35 (57.4)	59 (60.2)	99 (57.6)	0.72
Dyslipidaemia, *n* (%)	70 (40.9)	26 (42.6)	46 (46.9)	75 (43.6)	0.25
Diabetes, *n* (%)	48 (28.1)	18 (29.5)	32 (32.7)	58 (33.7)	0.52
Previous smokers, *n* (%)	54 (31.6)	22 (36.1)	35 (35.7)	57 (33.3)	0.62
Active smokers, *n* (%)	31 (18.1)	10 (16.4)	14 (14.3)	27 (15.7)	0.18
Valvular heart disease, *n* (%)	37 (21.6)	16 (26.2)	23 (23.5)	40 (23.2)	0.72
LVEF (%)	52.5 ± 10.7	54.1 ± 8.9	52.6 ± 10.1	53.1 ± 9.7	0.32
LA diameter (mm)	30.1 ± 7.3	31.6 ± 6.9	29.2 ± 7.8	30.3 ± 7.4	0.78
CCS class	2.7 ± 1.1	2.4 ± 0.8	2.5 ± 0.9	2.6 ± 0.9	0.59
TIA/stroke, *n* (%)	46 (26.9)	19 (31.1)	32 (32.7)	50 (29.1)	0.61
eGFR <30 mL/min/1.73 m^2^	27 (15.8)	11 (18)	16 (16.3)	42 (17.7)	0.55
HF, *n* (%)	60 (35.1)	21 (34.4)	30 (30.6)	60 (34.8)	0.68
COPD, *n* (%)	50 (29.2)	19 (31.1)	32 (32.7)	58 (33.7)	0.72
PAD, *n* (%)	42 (24.6)	13 (21.3)	18 (18.4)	39 (22.6)	0.25
EUROSCORE	20.3 ± 9.4	20.2 ± 9.3	23.1 ± 10.5	24.5 ± 10.1 *	0.02
Clinical presentation					
Silent ischemia	5 (2.9)	2 (3.3)	5 (5.1)	7 (4.1)	0.55
N-STEMI, *n* (%)	78 (45.6)	26 (42.6)	40 (40.8)	73 (42.4)	0.68
Unstable angina, *n* (%)	77 (45.0)	27 (44.3)	49 (50)	75 (43.6)	0.87
Recent STEMI (>24 h)	21 (12.3)	8 (13.1)	11 (11.2)	17 (9.8) *	0.58

**Table 2:** Lesion and procedural characteristics of the analyzed cohorts of patients. * Defined as moderate calcification (radiopaque densities noted only during the cardiac cycle and typically involving only 1 side of the vascular wall) or severe calcification (radiopaque densities noted without cardiac motion before contrast injection and generally involving both sides of the arterial wall). LAD: left anterior descending coronary artery; LCx: left circumflex coronary artery; LM: left main; RCA: right coronary artery. * *p* < 0.05 NIT vs. crossover; ** *p* < 0.05 NIT vs. T or TAP.

	Crossover *n* = 171	T or TAP *n* = 61	Culotte *n* = 98	NIT *n* = 172	*p*
Three-vessel disease	101 (59.6)	34 (55.7)	67 (68.4)	132 (76.8)	0.01
LM lesion location					
Ostial, *n* (%)	28 (16.3)	10 (16.4)	17 (17.3)	38 (22.0)	0.01
Body shaft, *n* (%)	34 (19.9)	17 (27.8) **	37 (37.7)	69 (40.1) *	0.02
Distal LM, *n* (%)	171 (100)	61 (100)	98 (100)	172 (100.0)	0.99
Medina 1,1,1 bifurcation, *n* (%)	74 (43.2)	30 (49.1)	41 (41.8)	83 (48.2)	0.55
Medina 0,1,1 bifurcation, *n* (%)	51 (29.8)	18 (29.5)	30 (30.6)	45 (26.1)	0.65
Trifurcation, *n* (%)	46 (35.0)	13 (21.3)	27 (27.5)	44 (25.5) *	0.52
Calcification *, *n* (%)					
Moderate, *n* (%)	18 (10.5)	11 (18.0)	17 (17.3%)	34 (19.7) *	0.39
Severe, *n* (%)	15 (8.7)	9 (14.7)	13 (13.2%)	30 (17.4) *	0.55
Chronic total occlusion	37 (21.6)	9 (14.7)	13 (13.2)	33 (19.8)	0.65
LM, *n*	1	0	0	1	-
LAD, *n*	13	2	8	10	-
LCx, *n*	19	3	0	13	-
RCA, *n*	4	4	5	9	-
TIMI flow grade < 3					
Main vessel	15 (8.7)	6 (9.8)	7 (7.1)	12 (6.8)	0.66
Side branch	18 (10.5)	5 (8.1)	8 (8.1)	16 (9.3)	0.59
Syntax	28.8 ± 8.1	29.1 ± 7.6	30.3 ± 7.0	31.6 ± 6.3 *	0.02
Stent characteristics					
Mean LM stent diameter (mm)	4.3 ± 0.8	4.3 ± 0.7	4.4 ± 0.8	4.5 ± 0.9	0.60
Mean number of stent	1.5 ± 0.5	2.2 ± 0.5	2.5 ± 0.5	2.8 ± 0.4	0.02
Global stent length (mm)	26.8 ± 10	33.8 ± 10	46.1 ± 11	46.4 ± 10	0.02

**Table 3:** Pattern of positivity of 6-month exercise stress tests. ECG: electrocardiogram; NIT: nano-inverted-T, T; T-stenting; TAP: T and protruding.

Single Stent		Double-Stent Strategy	*p*
Strategy			
	*n* = 171 (%)	*n* = 331 (%)	
Negative	154 (90)	269 (81.2)	0.01
Inconclusive	10 (5.8)	27 (8.1)	0.34
aVr ST elevation > 1 mm	1 (0.6)	12 (3.6)	0.04
V3-V6 ST depression > 1 mm	4 (2.3)	25 (7.5)	0.01
DIII-aVf depression/elevation > 1 mm	1 (0.6)	3 (0.9)	0.72
ECG only during the stress tests	2 (1.2)	19 (5.7)	0.01
Symptom only during the stress tests	2 (1.2)	4 (1.2)	0.99
ECG + Symptoms during the stress tests	3 (1.8)	16 (4.8)	0.09

**Table 4:** Clinical, anatomical, procedural parameters and clinical outcomes distribution among patients with and without positive 6-month exercise stress test. CV: cardiovascular; GFR: glomerular filtration rate; TLF: target lesion failure; TLR: target lesion revascularization; ST: stent thrombosis.

Six-Month Exercise Stress Test			
	Positive *n* = 42 (%)	Negative *n* = 460 (%)	*p*
Gender (females)	12 (28.5)	174 (37.8)	0.23
Age ≥ 75 years	10 (23.8)	191 (41.5)	0.02
Obesity	5 (11.9)	73 (15.9)	0.49
Diabetes	17 (40.5)	139 (30.2)	0.16
Dyslipidemia	29 (69)	188 (40.9)	<0.001
eGFR < 30 mL/min/1.73 m^2^	3 (7.1)	93 (20.2)	0.03
Triple-vessel disease	38 (90.5)	296 (64.3)	<0.001
Additional ostial LM lesion	36 (85.7)	57 (12.4)	<0.001
Additional body LM lesion	39 (92.8)	118 (25.6)	<0.001
Syntax > 25	40 (95.2)	342 (74.3)	0.003
Use of Rotablator	8 (19)	6 (1.3)	<0.001
Mean number of stent	2.8 ± 0.5	2.0 ± 0.5	<0.001
Global stent length (mm)	33.7 ± 9	28.7 ± 11	0.04
TLF	34 (80.9)	17 (3.7)	<0.001
TLR	18 (97.9)	9 (1.9)	<0.001
ST	5 (11.9)	1 (0.2)	<0.001
CV Death	11 (26.2)	7 (1.5)	<0.001
Clinical restenosis	33 (78.5)	5 (1.1)	<0.001

## Data Availability

The data presented in this study are available on request from the corresponding author.

## Supplementary Materials

The following supporting information can be downloaded at: https://www.mdpi.com/article/10.3390/diagnostics14010059/s1, Supplementary File S1.
