# Supplementary material for: Prognostic Impact of a Routine Six-Month Exercise Stress Test after Complex Left Main Bifurcation Percutaneous Intervention
Source: Diagnostics (Basel). 2023 Dec 26;14(1):59. doi: 10.3390/diagnostics14010059 (PMC10795681; doi:10.3390/diagnostics14010059)
Supplement: Supplementary file 1 [file diagnostics-14-00059-s001.zip › diagnostics-2751854-supplementary.pdf]

Supplementary File S1

	T or TAP N=61 (%)	Culotte N=98 (%)	NIT N=172 (%)
Negative	45 (73.3)*	74 (75.5)	150(87.2)
Inconclusive	6(9.8)	8(8.1)	13(7.5)
aVr ST elevation > 1mm	2(3.3)	5 (5.1)	0
V3-V6 ST depression >1 mm	8 (13.1)*	8(8.1)	6(3.5)
DIII-aVf depression/elevation >1 mm	1(1.6)	2 (2)	0
ECG only during the stress tests	6(9.8)*	9(14.7)**	4(2.3)
Symptom only during the stress tests	2(3.3)	2(2)	0
ECG+Symptoms during the stress tests	2(3.3)	4 (4.1)	2(1.2)

**p<0.05 between NIT and T or TAP; **p<0.05 between NIT and Culotte*
